# Supplementary material for: Inhibiting Endothelial Cell Function in Normal and Tumor Angiogenesis Using BMP Type I Receptor Macrocyclic Kinase Inhibitors
Source: Cancers (Basel). 2021 Jun 12;13(12):2951. doi: 10.3390/cancers13122951 (PMC8231556; doi:10.3390/cancers13122951)
Supplement: Supplementary file 1 [file cancers-13-02951-s001.zip › cancers-1226985-supplementary.pdf]

cancers MDPI

Supplementary Material: Inhibiting Endothelial Cell Function in Normal and Tumor Angiogenesis Using BMP Type I Receptor Macrocyclic Kinase Inhibitors

Jin Ma, Jiang Ren, Midory Thorikay, Maarten van Dinther, Gonzalo Sanchez-Duffhues, Josselin Caradec, Pascal Benderitter, Jan Hoflack and Peter ten Dijke

Table S1. Primers used for qRT-PCR.

Gene	Forward	Reverse
GAPDH	AACTTTGGCATTGTGGAAGG	ACACATTGGGGGTAGGAACA
ALK1	TGCGGGAGGTCATGTCTGA	CTGGTTCCGGGAGACTGAGAT
ALK2	TCTGGTTCCGGGAGACTGAGAT	TGCGGGAGGTCATGTCTGA
ID1	CTGCTCTACGACATGAACGG	GAAGGTCCCTGATGTAGTCGAT
ID3	CACCTCCAGAACGCAGGTGCTG	AGGGCGAAGTTGGGGCCCAT
SMAD6	ACAAGCCACTGGATCTGTCC	ACATGCTGGCGTCTGAGAA
DLL4	TGCGAGAAGAAAGTGGACAG	ACAGTAGGTGCCCGTGAATC
NR4A	TGGACGGCTACACAGGAGAGT	AGGAGCATGGCTGGACTGTT

Table S2. Inhibitory effect (%) of OD16 and OD29 or LDN-193189 (at 0.1 μM) on a panel of protein kinases.

Protein Kinase	OD16 inhibition	OD29 inhibition	LDN−193189 inhibition
ABL1	−20.09	18.66	12.47
ACVR1	90.23	92.52	67.72
ACVR1B	7.78	5.71	23.95
AKT1	−4.07	−2.83	16.13
AURKB	4.48	−11.24	3.93
AXL	−34.49	5.83	10.68
BRAF	5.01	5.1	7.79
BTK	−12	7.22	−12.38
CAMK1D	−5.9	−7.41	4.59
CAMK2D	−3.48	9.77	−13.28
CAMKK1	−1.72	−29.53	0.77
CDK1_cyclinA2	−8.88	23.74	−2.18
CDK4_cyclinD1	20.29	−13.83	−10.13
CDK8_cyclinC	−16.6	−9.32	−1.99
CDK9_cyclinT1	54.2	−2.77	−26.68
CHEK1	0.85	4.25	−34.61
CHEK2	7.12	−0.87	−0.88
CLK2	−10.04	7.62	34.16
CSNK1A1	0.99	−2.21	−3.63
CSNK1G2	−15.55	−0.5	−23.11
CSNK2A1	−9.27	−1.02	−10.92
DAPK1	−6.59	−3.89	11.27
DCLK2	−2.18	−15.88	9.12
DDR2	33.48	12.89	10.13
DNAPK	−8.88	12.91	−29.65
DYRK1A	−6.26	−7.58	−15.62
EGFR	−12.97	−8.91	2.46
EIF2AK1	−7.88	−0.05	−16.27
EPHA2	−21.87	10.44	−17.18

EPHA5	−65.65	14.81	−0.32
EPHB4	−36.62	3.64	4.39
FER	−18.85	−7.91	14.4
FGFR3	14.75	49.53	4.55
FLT3	4.56	−4.23	3.07
FYN	−31.04	24.64	28.05
GRK6	−2.14	0.04	−1.81
GSG2	6.26	34.59	−5.75
GSK3B	1.33	3.34	−28.24
HIPK1	−7.23	6.23	−13.45
IGF1R	−39.45	13.89	8.78
IKBKB	1.32	−9.1	1.34
IRAK4	4.7	26.44	−6.28
JAK1	23.14	−3.74	−10.89
JAK2	50.2	14.43	5.83
KDR	0.8	37.56	−6.35
KIT	32.99	7.49	−20.32
LCK	6.7	35.64	25.43
LIMK1	30.29	1.15	8.92
LRRK2	18.39	13.85	5.16
MAP2K1	5.48	7.68	11.05
MAP3K10	1.85	1.57	−26.42
MAP3K5	−11.3	0.68	6.9
MAP3K7	−6.98	11.39	7.07
MAP3K8	−16.87	−5.03	−10.33
MAP4K4	−0.07	13.47	−4.01
MAP4K5	14.64	19.12	39.72
MAPK14	−3.1	45.19	0.3
MAPK3	−13.85	−7.55	−8.08
MAPK9	−10.96	5.44	−21.26
MAPKAPK2	−14.63	−4.8	0.06
MET	5.95	−3.42	1.47
NEK1	−6.3	12.59	−11.86
NEK6	−21.07	4.01	7.05
NTRK1	−37.92	69.6	14.06
NUAK1	74.31	5.32	−20.18
PAK2	−27.07	7.91	−2.66
PDGFRA	−15.96	−9.93	3.63
PDK1	−7.16	−1.4	−8.72
PHKG2	1.87	−1.93	−23.55
PIM2	−5.63	1.97	6.22
PLK1	−5.35	−21.25	−21.71
PLK4	41.4	4.1	−34.35
PRKACA	−19.52	9.09	−14.06
PRKCB1_II	15.9	3.7	1.8
PRKD2	32.16	5.45	−23.12
PTK2	17.09	−0.22	4.03
RET	−29.08	27.14	10.83
RIPK2	36.48	62.48	91.14
ROCK2	42.64	−0.26	13.08
RPS6KA1	17.02	5.09	−10.42
RPS6KB1	−14.96	−3.52	13.02
SGK3	2.44	−21.89	−26.87
SIK2	43.84	1.13	44.02
SLK	26.52	27.7	−18.43
SRC	−19.39	−4.82	2.89
SRPK1	−24.92	21.5	−16.47

STK4	−5.76	20.45	−21
TEK	−49.11	−6.29	38.36
TGFBR2	25.48	18.92	61.11
TNK2	6.34	−22.55	8.11
TSSK1B	12.5	−9.62	4.23
TTK	−2.28	3.83	9.07
VRK1	−11.85	−9.5	3.78
WEE1	−2.29	−0.95	1.81
WNK1	0.23	−7.33	2.52
ZAK	15.73	4.36	57.37

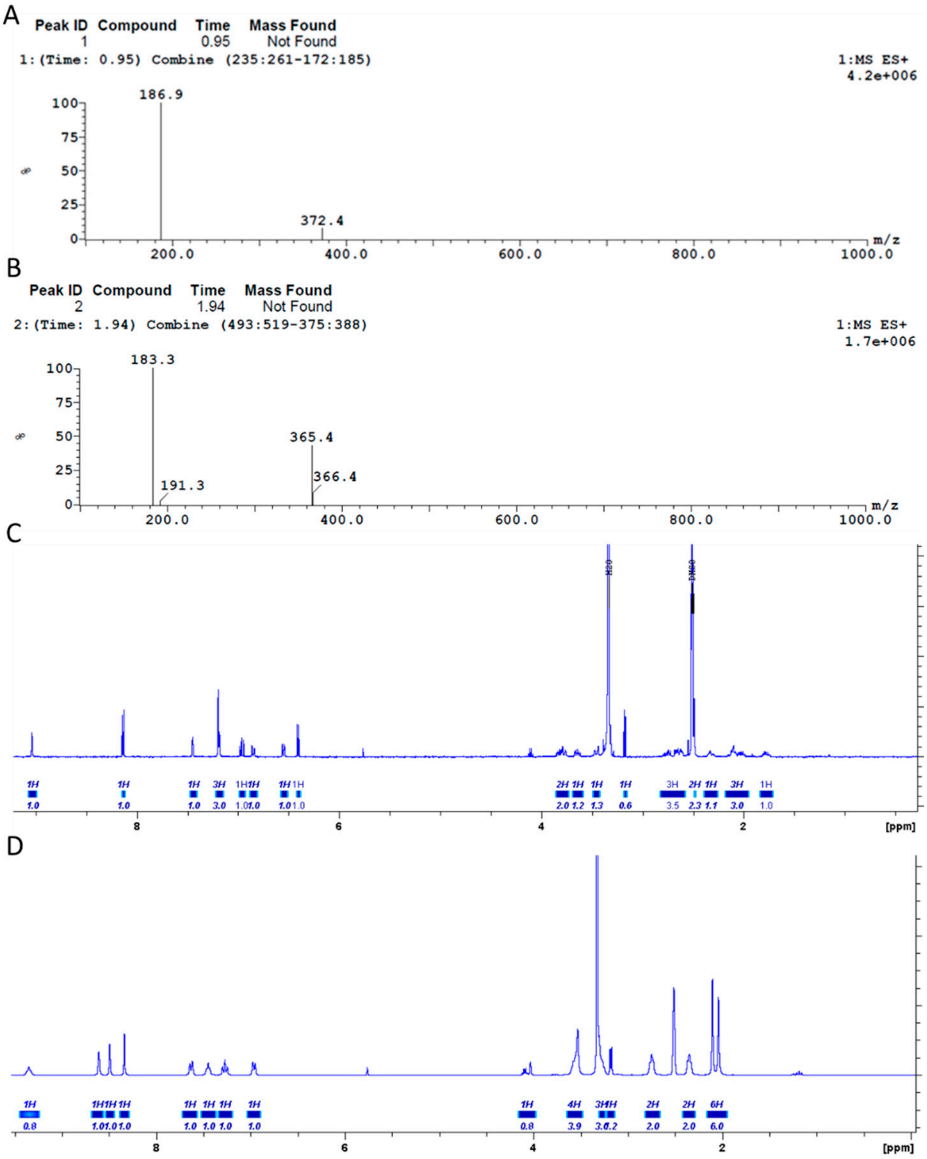

Figure S1. Characterization of OD16 and OD29. Liquid chromatography–mass spectrometry (LCMS) results of OD16 (**A**) and OD29 (**B**). ^{1}H-NMR (DMSO, 400 MHz) spectra of OD16 (**C**) and OD29 (**D**).

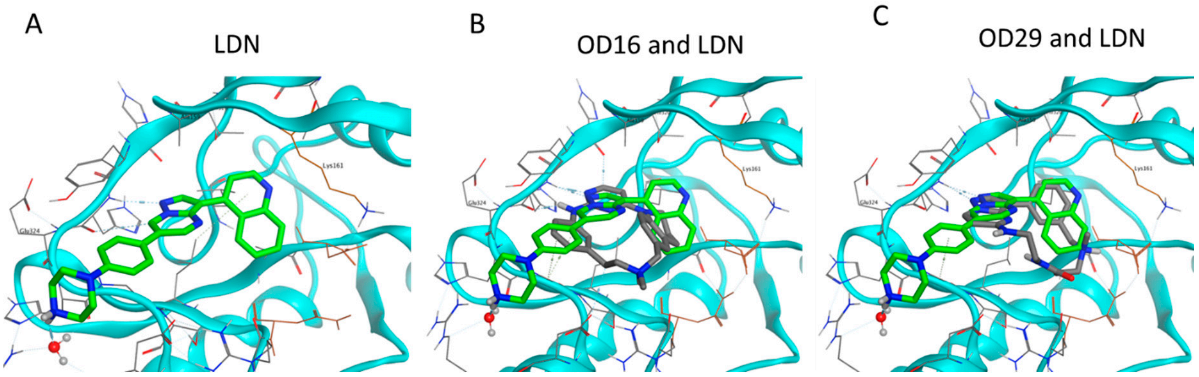

Figure S2. Structural model of (**A**) LDN−193189, (**B**) OD16 (grey) overlay with LDN−193189 (green), (**C**) OD29 (grey) overlay with LDN−193189 (green) bind to the ALK1 hinge region in the ATP pocket.

Gene	Cells	Mean Cq	Cq SEM
ALK1	EA.hy926	24.21	0.04366
ALK2	EA.hy926	23.96	0.03098
ALK3	EA.hy926	27.38	0.04660
ALK6	EA.hy926	29.48	0.10524
GAPDH	EA.hy926	16.78	0.07856
ALK1	HUVEC	24.55	0.08405
ALK2	HUVEC	25.87	0.07868
ALK3	HUVEC	39.65	0.00930
ALK6	HUVEC	33.27	0.46645
GAPDH	HUVEC	17.76	0.10185

Figure S3. RT-qPCR analysis of the *ALK1*, *ALK2* and *GAPDH* mRNA expression in EA.hy926 and HUVEC cells. Mean Cq and Cq standard error of the mean (SEM) are listed. Representative results from three independent experiments are shown.

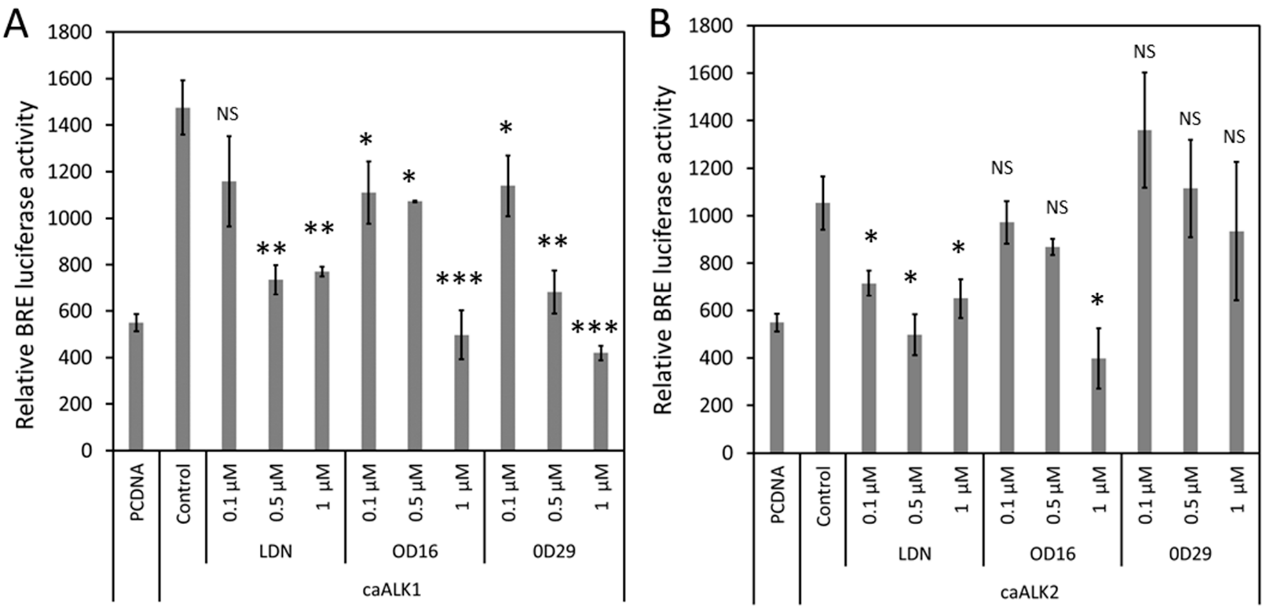

Figure S4. OD29 and OD16 are two small molecule BMP type I receptors kinase inhibitors in C2C12 cells. Effects of vehicle control (DMSO), LDN−193189, OD16 or OD29 on BRE transcriptional luciferase reporter activity induced by the ectopic expression of caALK1 (**A**) or caALK2 (**B**) in C2C12 cells. Cells were incubated with the inhibitors in three different concentrations (0.1 µM, 0.5 µM or 1 µM) for 16 hours. Representative results from three biologically independent experiments are shown as mean ± SD. NS, not significant; * $p < 0.05$, ** $p < 0.005$; *** $p < 0.001$.

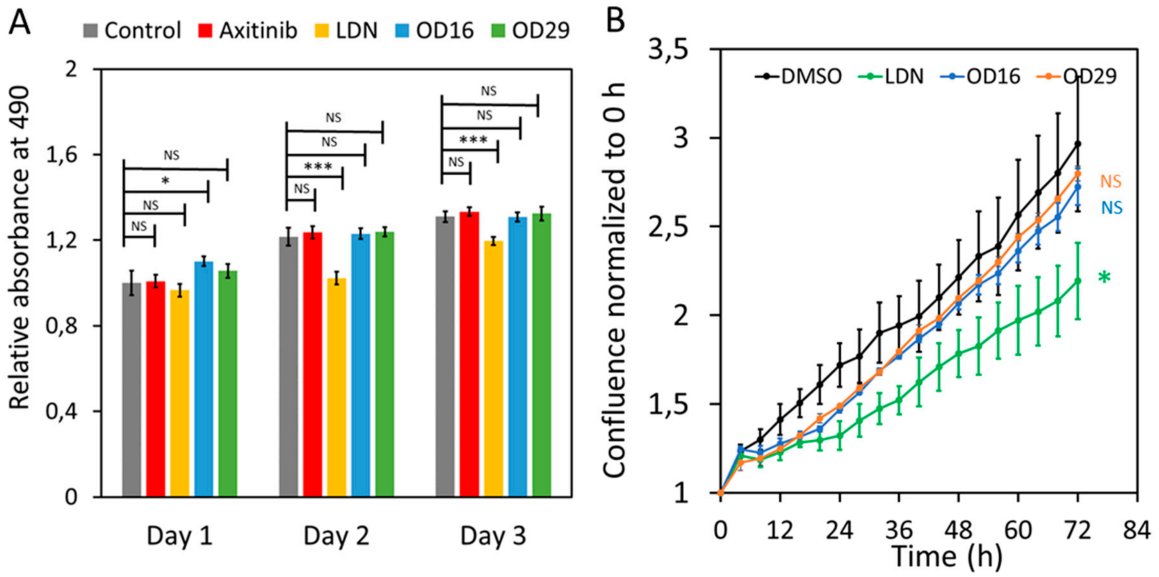

Figure S5. Effects of OD16 or OD29 on EA.hy926 cell proliferation. **(A)** The effects of vehicle control (DMSO), Axitinib, LDN−193189, OD16 or OD29 (0.5 µM) on EA.hy926 cell metabolic activity after day 1, day 2 and day 3 treatment as measured by MST assay. (**B**) The effects of vehicle control (DMSO), LDN−193189, OD16 or OD29 (0.5 µM) on EA.hy926 cell confluence during 3 days as monitored using IncuCyte. NS, not significant; * $p << 0.05$, *** $p < 0.001$.

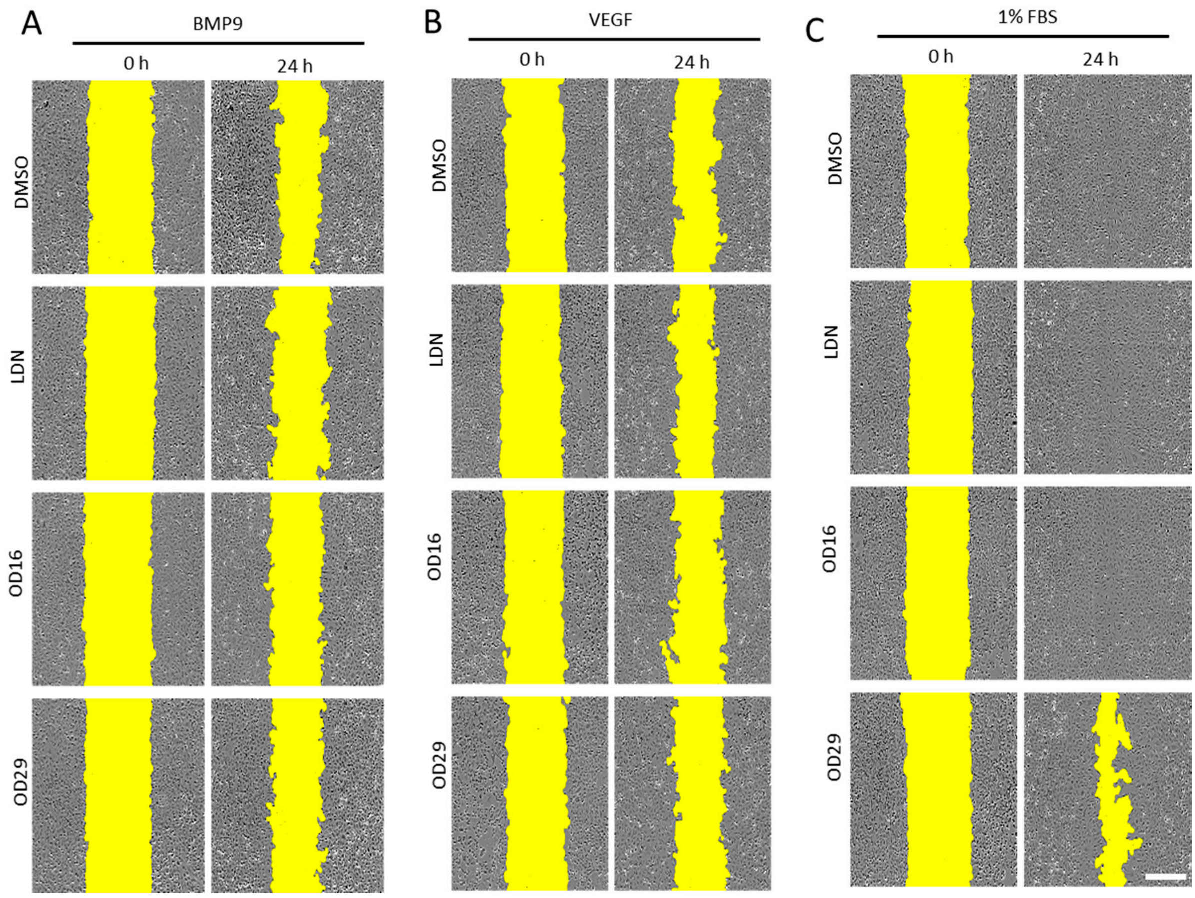

Figure S6. Effects of OD16 and OD29 on EA.hy926 cell migration. Representative images from 2D migration assay of EA.hy926 cells exposed to BMP9 (1 ng/mL) in the presence of vehicle control (DMSO), LDN−193189, OD16 or OD29 (0.5 µM) with (**A**) BMP9 (1 ng/mL), (**B**) VEGF (15 ng/mL) and (**C**) 1% FBS at 0 h and 24 h. The unclosed area is labelled in yellow. Scale bar represents 400 µm.

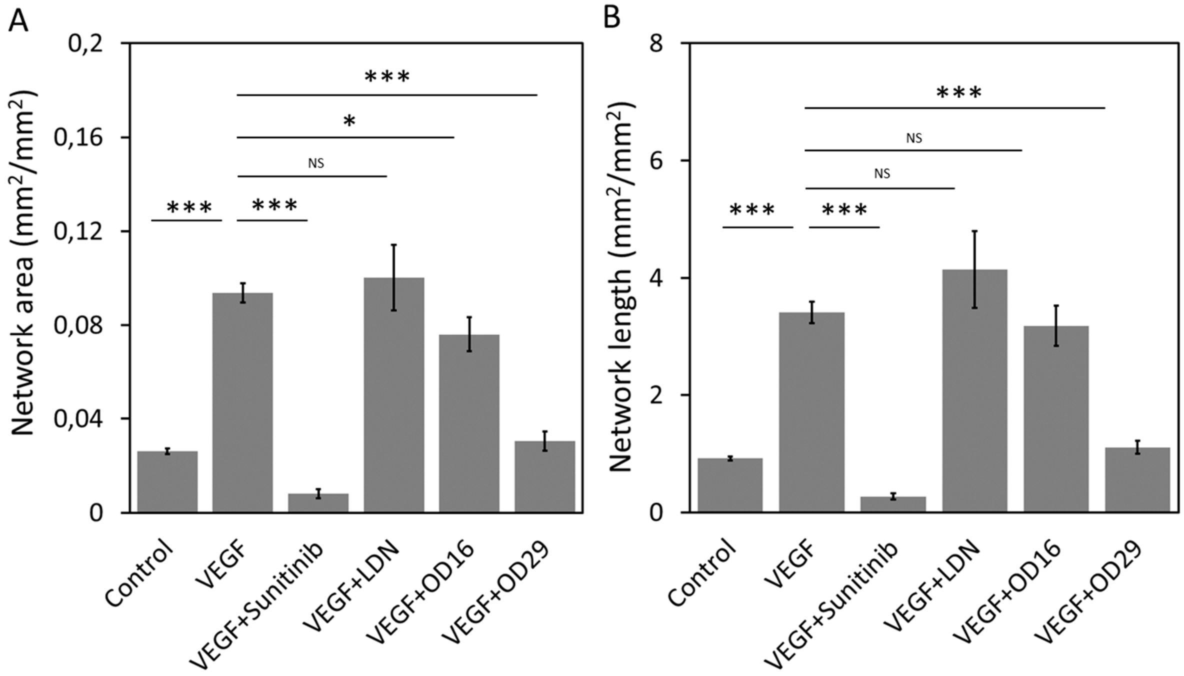

Figure S7. OD16 or OD29 attenuate EC cord formation. Cord formation assay was performed by co-culturing the HUVEC-eGFP cells with human dermal fibroblasts and stimulated with VEGF (15 ng/mL). Cells were co-cultured for 6 days to assess the effects of vehicle control (DMSO), OD16 or OD29 (0.5 μM). The VEGFR kinase inhibitor Sunitinib (1 μM) and LDN−193189 (0.5 μM) were included for comparison. The cord formation of each group at day 6 was quantified. (**A**) The network area is shown as mean ± SD. (**B**) The network length is shown as mean ± SD. Representative results from three biologically independent experiments are shown. NS, not significant; * $p < 0.05$; *** $p < 0.001$.

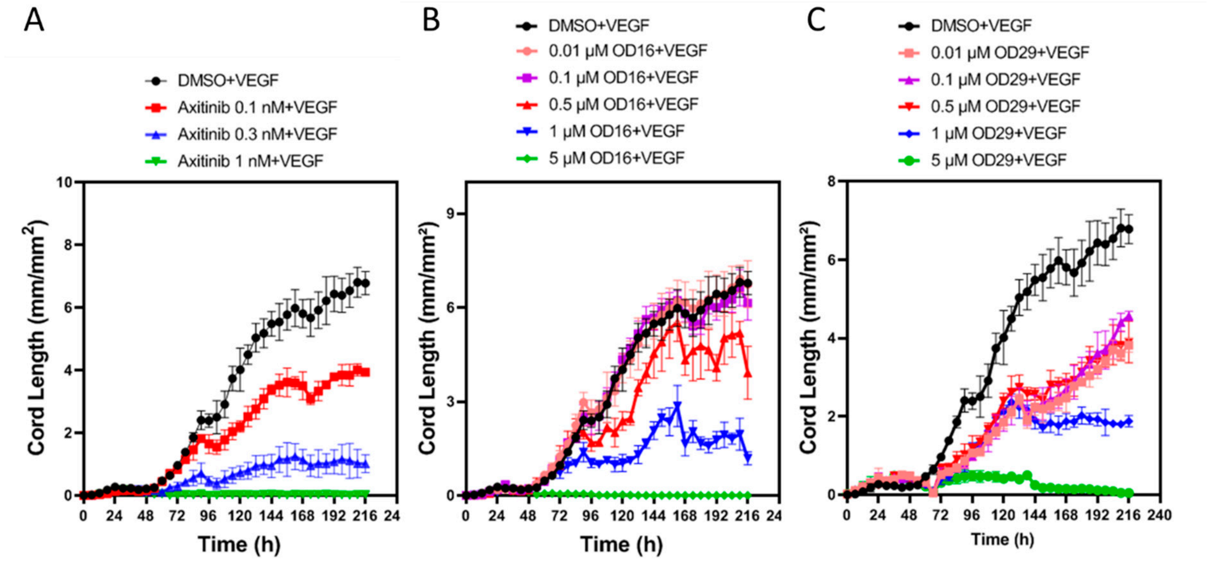

Figure S8. OD16 or OD29 attenuate EC cord formation in a dose-dependent manner. Cord formation assay was performed by co-culturing HUVEC-eGFP cells with human dermal fibroblasts and stimulated with VEGF (20 ng/mL). Cells were co-cultured for 9 days to assess the effects of vehicle control (DMSO), Axitinib (**A**) at different concentrations (0.1 nM, 0.3 nM and 1 nM), OD16 (**B**) or OD29 (**C**) at different concentrations (0.01 μM , 0.1 μM, 0.5 μM, 1 μM and 5 μM) on cord length. The cord length is shown as mean ± SD.

Figure 2A

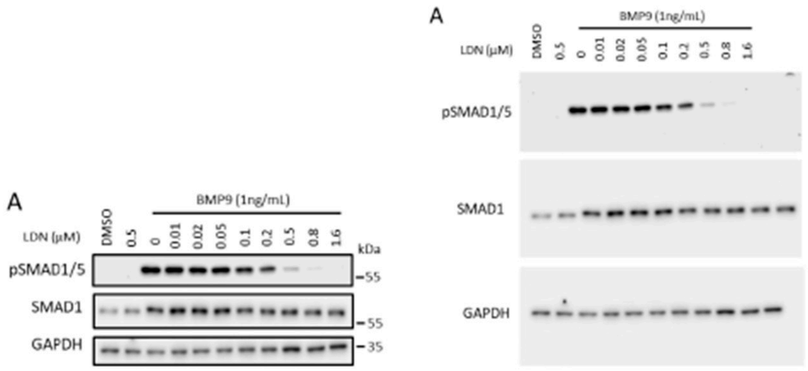

Figure 2B

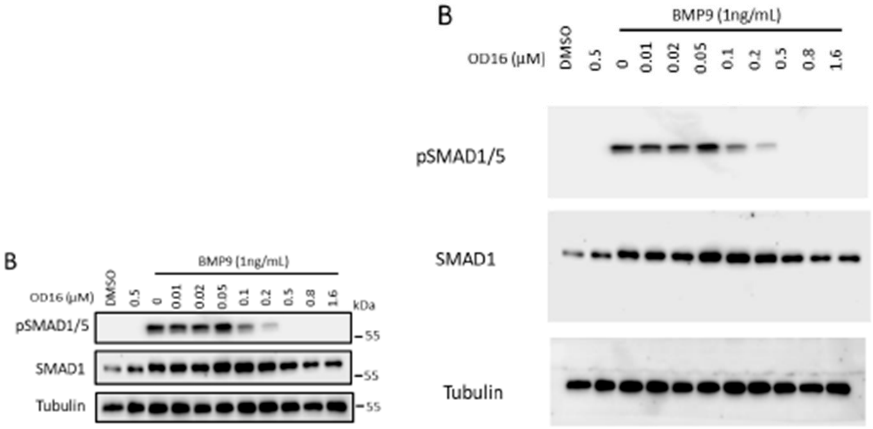

Figure 2C

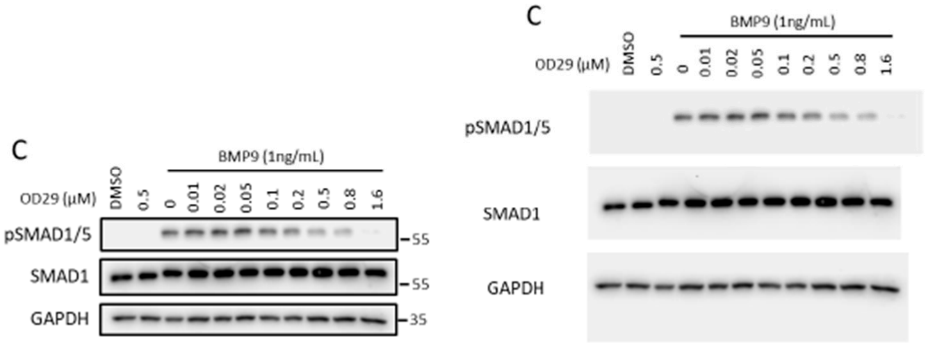

Figure 2J

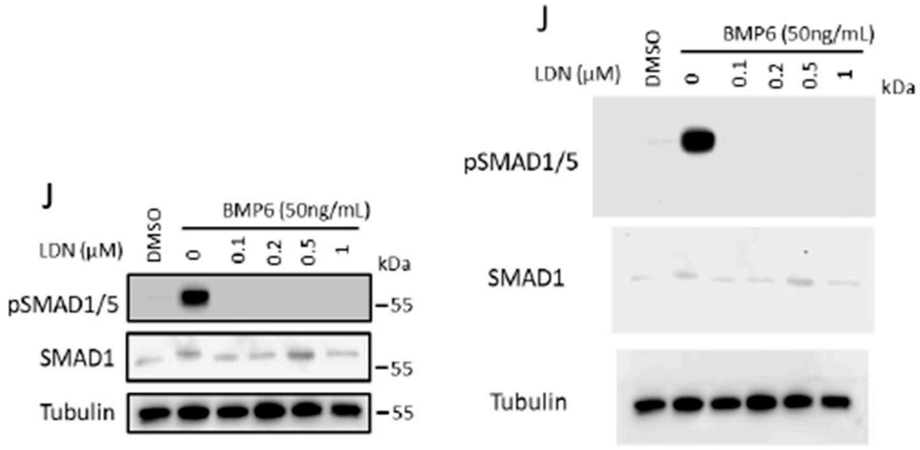

Figure 2K

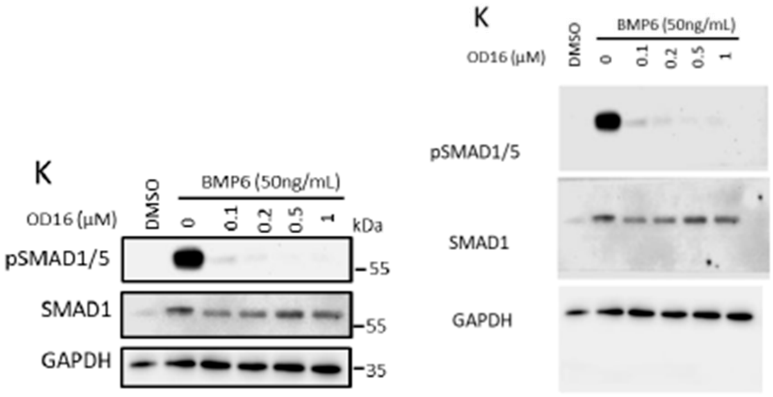

Figure 2L

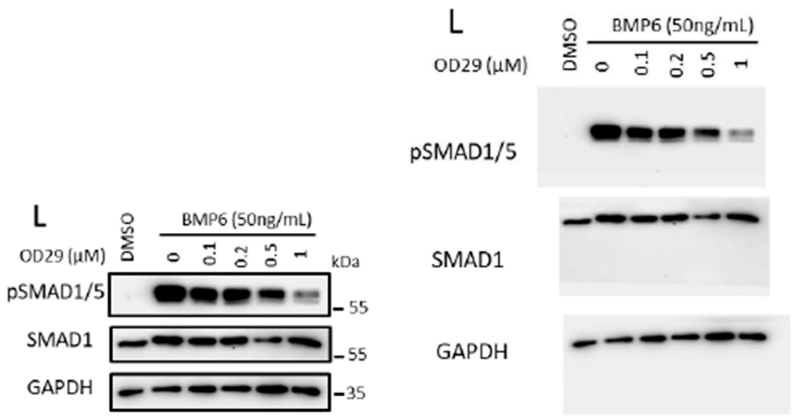

Figure 2N

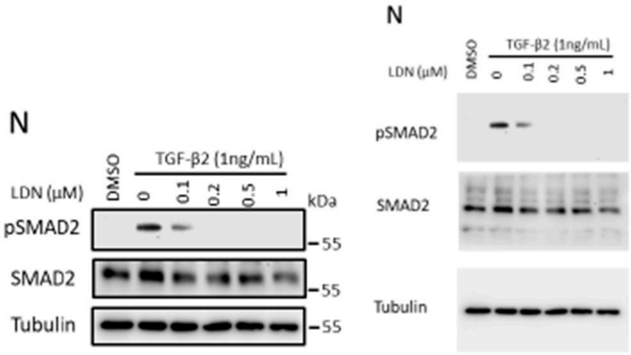

Figure 2O

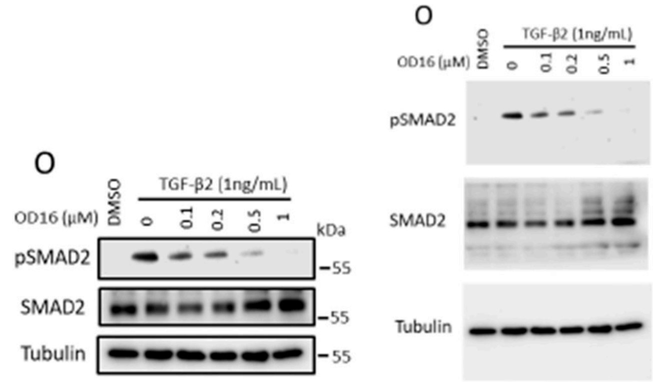

Figure 2P

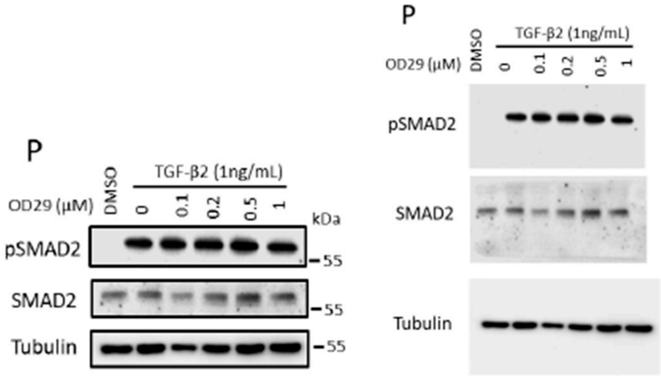

Figure S9. Complete Western Blot images of subfigures 2A–C and 2J, 2K, 2L, 2N, 2O, 2P.

Figure 3A

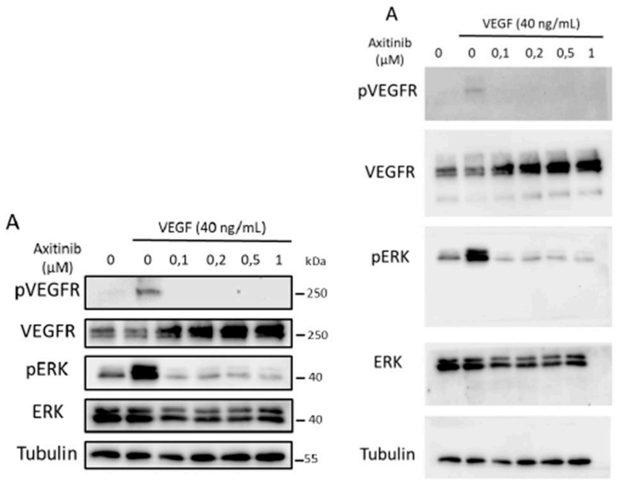

Figure 3B

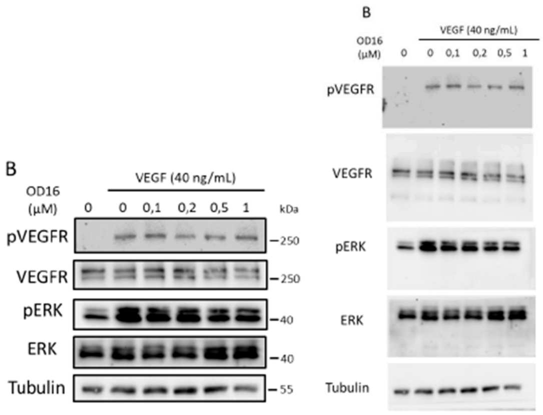

Figure 3C

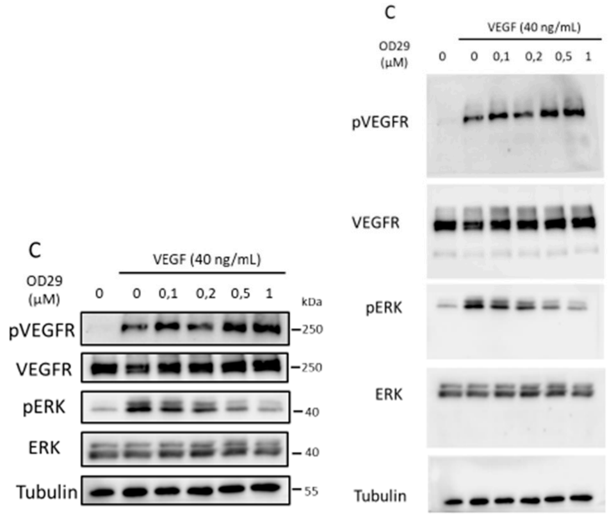

Figure S10. Complete Western Blot images of subfigures 3A–C.
